# Use of Placebo in Supplementation Studies—Vitamin D Research Illustrates an Ethical Quandary

**DOI:** 10.3390/nu10030347

**Published:** 2018-03-13

**Authors:** Leigh A. Frame, Jonathan P. Fischer, Gail Geller, Lawrence J. Cheskin

**Affiliations:** 1 The George Washington School of Medicine and Health Sciences, Washington, DC 20052, USA; 2 The University of Chicago Pritzker School of Medicine, Chicago, IL 60637, USA; jonf@uchicago.edu; 3 The Johns Hopkins School of Medicine, Baltimore, MD 21205, USA; ggeller@jhu.edu; 4 The Johns Hopkins Bloomberg School of Public Health, Baltimore, MD 21205, USA; cheskin@jhu.edu; 5 The Johns Hopkins Berman Institute of Bioethics, Baltimore, MD 21205, USA; 6 The Johns Hopkins Weight Management Center, Baltimore, MD 21205, USA; 7 The Global Obesity Prevention Center at Johns Hopkins, Baltimore, MD 21205, USA

**Keywords:** nutrition therapy, dietary supplements, research ethics, research design, vitamin D

## Abstract

History has shown that without explicit and enforced guidelines, even well-intentioned researchers can fail to adequately examine the ethical pros and cons of study design choices. One area in which consensus does not yet exist is the use of placebo groups in vitamin supplementation studies. As a prime example, we focus on vitamin D research. We aim to provide an overview of the ethical issues in placebo-controlled studies and guide future discussion about the ethical use of placebo groups. Research in the field of vitamin D shows variation in how placebo groups are used. We outline four types of control groups in use: active-control, placebo-control with restrictions on supplementation, placebo-control without supplementation restrictions, and placebo-control with rescue repletion therapy. The first two types highlight discrete ethical issues: active-control trials limit the ability to detect a difference; placebo-control trials that restrict supplementation potentially place subjects at risk of undue harm. The final two, placebo-control without supplementation restrictions or with rescue repletion therapy, offer potential solutions to these ethical challenges. Building on this, guidelines should be established and enforced on the use of placebo in supplementation studies. Furthermore, the field of vitamin D research has the potential to set an example worthy of emulation.

## 1. Introduction

The field of research ethics has advanced remarkably in the past century. From deliberately infecting poor immigrants with yellow fever in the early 1900s, to the atrocities of Nazi “research” during World War II, to observing the natural progression of untreated syphilis among African-American sharecroppers and withholding effective treatment from them, human research has grown to be much more humane, driven and accompanied by the development of the field of research ethics. In spite of this important growth, unanswered ethical questions remain within research. Indeed, ethical issues will likely persist, as researchers struggle to balance conflicting interests and priorities. It is, however, vitally important that these ethical questions be actively discussed and consensus built around proper conduct. History has shown repeatedly that without explicit and enforced guidelines, even well-intentioned researchers can fail to adequately examine the ethical pros and cons of study design choices. 

One area in which consensus does not yet exist is the use of placebos in vitamin supplementation studies. As a prime example of such issues, we focus on vitamin D research. This paper aims to provide an overview of the ethical issues involved in placebo-controlled studies in vitamin D research, and guide future discussion about the ethical use of placebo groups.

## 2. Background

World War II marked a significant shift in medical ethics. In 1947, following the uncovering of atrocities committed by Nazi physicians performing research on prisoners, the Nuremberg Code was devised to provide guidelines on appropriate treatment of research subjects [1,2]. It outlined principles like obtaining consent, minimizing risk, and banning forced experimentation. 

Subsequently, however, many scientists felt that the Nuremberg Code alone was not specific enough [3]. For example, no mention was made of research involving vulnerable populations. The World Medical Congress created a more exhaustive research standard called the Declaration of Helsinki, in 1964 [3].

Despite the existence of these two documents, grossly unethical research continued. In 1966, an article by Henry Beecher described recent studies in which inhumane methods were employed, causing deliberate harm [4]. Beecher’s article highlighted that simply creating ethical standards was not enough—oversight and accountability were necessary.

In 1972, details of the now-infamous Tuskegee Syphilis Study were exposed. Effective treatment for syphilis infection was deliberately withheld from hundreds of poor African-American farmers without their knowledge [5].

Following the public outcry from the Tuskegee Syphilis Study and Beecher’s article, Congress passed the National Research Act (Pub. L. 93-348). This act established Institutional Review Boards for actively regulating ethics in all human subjects of medical research. 

The Belmont Report [6] added that all such research should adhere to the principles of respect for persons, beneficence, and justice. Many of its principles have been codified into US federal regulations. The 1991 Basic Policy for the Protection of Human Subjects (known as the Common Rule) is one such example [6].

Many of the ethical questions that arise today can be addressed by these documents. In areas where ethical ideals conflict, however, debate has continued within the research community.

## 3. Placebo-Controlled Studies

Since the mid-twentieth century, the use of placebos has gained traction, reaching gold-standard status in clinical research. Although questions remain about the mechanisms of the placebo effect, its potency is no longer in question [7,8]. Many, however, have questioned the ethics of placebo groups, weighing their benefits against possible increased risks [9]. In response, the *Declaration of Helsinki* stated in its 2000 revision that “In any medical study, every patient—including those of a control group, if any—should be assured of the best proven diagnostic and therapeutic method.” [10]. Elsewhere, it also stated that: “The benefits, risks, burdens and effectiveness of a new method should be tested against those of the best current prophylactic, diagnostic, and therapeutic methods. This does not exclude the use of placebo, or no treatment, in studies where no proven prophylactic, diagnostic or therapeutic method exists.” [10].

These statements provided a clear and concise guideline upon which placebo-controlled studies could be evaluated. However, the US Food and Drug Administration (FDA) was in support of the use of placebos in clinical trials even when effective treatments already exist [11]. In response to this discrepancy, a Note of Clarification was added in 2001 to the Declaration of Helsinki: “A placebo-controlled trial may be ethically acceptable, even if proven therapy is available, under the following circumstances: [w]here for compelling and scientifically sound methodological reasons its use is necessary to determine the efficacy or safety of a prophylactic, diagnostic or therapeutic method; or [w]here a prophylactic, diagnostic or therapeutic method is being investigated for a minor condition and the patients who receive placebo will not be subject to any additional risk of serious or irreversible harm.” [10].

This statement raised new questions. Some believed it caused the field of research ethics to move in the wrong direction, decreasing focus on the safety of individual participants [11]. Others, however, asserted that strict adherence to the 2000 Declaration of Helsinki would have hindered medical care by preventing effective evaluation of new treatments [12]. As such, there remained a lack of clear guidance over the ethics of placebos, and debate has continued. A review article by Flynn in 2003 described studies that, although arguably in compliance with the Declaration of Helsinki’s revised stance on placebos, resulted in adverse health events in participants assigned to placebo and were labeled unethical by the research community [13]. Thus, the need for discussion is still pertinent.

Vitamin D research today has placed too weak an emphasis on building consensus around the ethics of placebo-controlled studies, particularly in cases of known deficiency. It is imperative that an open debate exists on this topic. Doing so can lead to the formation of consensus, global enforcement of equivalent standards, and thorough justification of the ethics of all studies conducted in this field.

## 4. Vitamin D

Vitamin D is unique in the world of nutrition. It is a steroid hormone produced in the skin during exposure to the sun’s ultraviolet-B (UVB) radiation, and there are limited dietary sources of vitamin D. Historically, bone health has been the primary indication for administering oral vitamin D, which was discovered to prevent rickets, a softening of the bones, typically in children. More recently, vitamin D has been linked to a wide variety of health outcomes and is now classified as an immune-modulatory hormone. The potential adverse effects of withholding vitamin D supplementation in known cases of deficiency may include diminished bone health (short or long term) as well as increased risk of infection, autoimmunity, cancer, chronic disease, or even mental illness [14]. With our current knowledge, it is difficult to assess the precise risk for many of these potential risks, making the ethical quandary of placebo use in vitamin D supplementation studies all the more complex.

## 5. Vitamin D Research

Research in the field of vitamin D currently shows variation in how placebos are used, generating different ethical questions. The studies described below provide examples upon which a fruitful ethical discussion can be based.

### 5.1. Active Control: No Placebo Group

Numerous vitamin D studies have been conducted without a placebo group. This is especially common in vulnerable populations. For example, in 2011, Hollis et al. conducted a randomized controlled trial of vitamin D supplementation during pregnancy, comparing 400, 2000, and 4000 IU vitamin D_3_ daily in 350 pregnant women from 16 to 18 weeks gestation until delivery [15]. The Institute of Medicine estimated average requirement (average intake predicted to meet the need of 50% of the population) was 400 IU, which was considered minimal supplementation and used as a control group. Mean 25-hydroxyvitamin D (25(OH)D) concentrations (vitamin D status) were significantly different between groups one month prior to delivery and at the time of delivery; the highest status was achieved in the 4000 IU group with safety outcomes similar to the 400 IU group. This study is an example of where an active control group minimized ethical issues associated with use of placebos and yielded significant results. In 2013, Wagner et al. compared outcomes between pregnant women receiving 2000 and 4000 IU vitamin D_3_ daily [16]. The trajectories of 25(OH)D increase were significantly different (*p* < 0.01), with 4000 IU being steeper (faster improvement), but the final 25(OH)D concentrations were not significantly different (2000 IU: 90.1 nmol/L, 4000 IU: 94.4 nmol/L, *p* = 0.29). While there was a trend for reduced risk of infection, preterm labor, and preterm birth, these outcomes were not sufficiently powered, perhaps due to lack of a placebo group.

Although valuable information was gathered from these studies, some results were non-significant. The Belmont Report outlined the notion that researchers should strive to maximize the benefits of their research while minimizing potential risks [17]. When placebos are not used, it can be more difficult to observe significant results due to a reduced effect size between groups [18]. As such, the potential benefit of a research study can be diminished if lack of placebo leads to non-significant results. It would then be less ethical to expose participants to risk, even if minor, when it is unlikely for the study as designed to show significant benefits. 

This argument is especially relevant to vitamin D studies. Lappe and Heaney described this occurrence within vitamin D studies [18]. Vitamin D has been shown to exhibit a sigmoidal dose-response curve. This curve shows a small effect when changes are made to very low or very high intakes of vitamin D, and a larger effect when changes are made to intakes between these extremes. With an active-control study design, it is possible that both the treatment and control groups rest on an area of the curve that displays a small dose-response. This could explain why some of the results of Wagner et al. were non-significant, as they compared outcomes between two relatively high doses of vitamin D.

Thus, vitamin D studies may fit the qualifications outlined in the 2001 Note of Clarification of the Declaration of Helsinki for ethical uses of placebo-controlled studies discussed above. Placebos can be methodologically necessary, and further withholding supplementation may be unlikely to result in serious or irreversible harm. The risks related to short term poor vitamin D status, instead, are related to optimization of health and prevention of illness; for example, vitamin D supplementation may be prescribed to prevent falls in the elderly, slow the development of cardiovascular disease over decades, or decrease the attack rate during influenza season. This then raises the question of whether it is more ethical to include placebos to assure that the benefits of a study justify possible risks.

### 5.2. Placebo Group with Supplementation Restrictions

Some studies have used a randomized, placebo-controlled design and prohibited participants from consuming supplemental vitamin D. This is often considered the gold-standard method. Vitamin D supplementation in Older Subjects (ViDOS) is one such study [19,20]. ViDOS was a 1-year study in which 273 postmenopausal women with vitamin D deficiency were given placebo or one of seven doses of vitamin D (400, 800, 1600, 2400, 3200, and 4800 IU D_3_) in order to determine the dose that would yield 25(OH)D ≥75 nmol/L. Participants were prohibited from consuming additional vitamin D supplements. ViDOS has furthered the understanding of the dose response of vitamin D and explored the effect of race (stratified by Caucasian and African-American). In 2013, Roth et al. conducted a study of high-dose vitamin D_3_ in pregnant women (18 to 35 years of age, 26 to 29 weeks gestation) in an under-resourced area of Bangladesh [21]. Only women who consumed less than 400 IU vitamin D per day were included. Participants (*n* = 160) were randomized to receive either placebo or 35,000 IU vitamin D_3_ weekly until delivery. Participants were advised to abstain from additional vitamin D supplementation during the study, which averaged about 10 weeks. They found that 35,000 IU vitamin D_3_ weekly significantly increased 25(OH)D concentrations in mothers (134 vs. 38 nmol/L, *p* < 0.001) and umbilical cord blood (103 vs. 39 nmol/L, *p* < 0.001). Major pregnancy outcomes were similar between intervention and control groups.

The use of placebos in this fashion yielded valuable information, but raises ethical questions not present in studies that did not use placebos. Since vitamin D has a known benefit and vitamin D deficiency a known harm, it can be argued that withholding adequate supplementation poses an undue health risk to participants, especially those with known deficiency.

The potential benefits of understanding the ideal dose of vitamin D may not outweigh the possible harm being inflicted on participants who are withheld vitamin D supplementation. Further, since the effects of vitamin D deficiency are not yet fully understood, it is unknown whether or not such deficiency could cause serious or irreversible harm. The argument can be made, then, that based on the qualifications in the 2001 Note of Clarification of the Declaration of Helsinki, placebos should not be used in vitamin D studies.

The ViDOS study required vitamin D deficient participants to abstain from supplementation for one year. Those in the placebo group received insufficient amounts of vitamin D as a result of their participation in this study. The study by Roth et al. was conducted in an area where vitamin supplements are uncommon, so it likely did not cause any new risks to the vitamin D deficient pregnant women in the placebo group since they would not have supplemented otherwise. These researchers did, however, knowingly withhold effective treatment [21]. Both of these cases raise ethical questions over the appropriate use of placebos in their studies.

The study conducted by Roth et al. raises another unique question, as this study was conducted in an area where many people lack access to basic medical care [21]. Benjamin Freedman, in an article published in 1990, stated that if a healthcare system does not provide a minimal standard of care, a placebo-controlled study cannot be justified on the grounds that participants would not otherwise be receiving care [12]. In this case, an active-control group could have been used in which participants were given the Recommended Dietary Allowance (RDA), thus employing a low-dose versus high-dose design. Doing otherwise raises ethical concerns not only of imposing undue risk on participants, but also of misuse of participants from at-risk groups, which is a contradiction to the Belmont Report’s principle of justice [17]. 

## 6. Potential Solutions to These Ethical Issues

### 6.1. Placebo Group without Supplementation Restrictions

It is possible to utilize a randomized, placebo-controlled design but allow both treatment and control groups to consume supplemental vitamin D if desired. In 2011, Manson et al. published preliminary results from a large cohort study of vitamin D status, supplementation, and health outcomes in older US adults—VITamin D and OmegA-3 TriaL (VITAL) [22]. They randomized participants to placebo (no supplementation) or treatment (2000 IU vitamin D_3_ daily). The researchers state that all participants were allowed to supplement with vitamin D up to the RDA at the time, 400 IU per day, to ensure the safety of those in the placebo group, assuming that the level of voluntary intake would be similar between groups. Final results from VITAL are expected in shortly.

This study provides an example of using placebos while avoiding some of the ethical concerns found in other placebo-controlled studies. By allowing participants in both groups to take additional vitamin D supplementation, Manson et al. reduced the possibility of participation causing harm from vitamin D deficiency. Additionally, by measuring serum 25(OH)D concentration in all participants, they were still able to include an effective control group, albeit one that may have had slightly higher background 25(OH)D concentrations [22]. Although sound methodological justifications exist for requiring participants to abstain from additional vitamin D supplementation, allowing participants to consume additional vitamin D supplements addresses to some extent the ethical concerns raised by not doing so. Purists may argue that such an approach, while partially addressing the ethical concern, still does not treat all groups up to standard-of-care, as the participants most likely to take additional supplements are those with better access to care and/or with more education or higher socioeconomic status.

### 6.2. Placebo Group with Rescue Repletion

Another alternative worthy of discussion is to design a study that includes a placebo arm so that subsequent “rescue” treatment is routinely given to subjects. This approach may or may not reduce risks adequately, depending on the nature of the outcome being studied, but offers all subjects eventual gold-standard treatment, irrespective of their having the knowledge or caregiver adequacy to receive gold-standard care. Although participants in the placebo group may experience vitamin D deficiency during the course of the study, the rescue therapy reduces the risk of any serious or irreversible harm caused by deficiency. Such a design would then fit the Declaration of Helsinki’s qualifications for an ethical use of a placebo group. This design provides an exciting alternative, especially to studies conducted in under-resourced areas. To our knowledge, this approach to the ethical problems inherent in placebo-controlled trials has not yet been utilized in vitamin D studies, although it has in other areas of investigation such as pain management [23,24], nausea and vomiting [25], depressive disorder [26,27], and asthma [28]. In these studies, need for rescue therapy is used as a marker of treatment failure.

## 7. Conclusions

The field of vitamin D research lies at a crossroads. It is imperative that researchers in this field ensure ethical considerations are given a high priority in study design. We outlined four types of control groups used in supplementation studies: active-control, placebo-control with restrictions on vitamin D supplementation, placebo-control without supplementation restrictions, and placebo-control with rescue repletion therapy. The first two types highlight discrete ethical issues: active-control trials limit the ability to detect a difference; placebo-control trials that restrict supplementation potentially place subjects at risk of undue harm. The final two, placebo-control without supplementation restrictions or with rescue repletion therapy, offer potential solutions to these ethical challenges. 

The nutrition research community should establish guidelines for the ethical use of placebo-controlled supplementation studies. Before placebos are used in a study, investigators should engage in robust discussion over the ethical implications. These are often a function of study design issues such as sample size and power [29,30]. Nutrition researchers should understand the relationship between ethical and methodologic challenges. Arguably, it is unethical to enroll any participants in a clinical trial that is not powered to detect a difference. Journal editors and funding agencies should also demand thorough ethical justifications of the use of placebos, especially in cases of known vitamin D deficiency or in vulnerable populations. Incredible advances have taken place in the ethical oversight of human research subjects over the past several decades. It is crucial that this work continue so that guidelines are both established and enforced on the use of placebo groups in vitamin D research (as well as potentially a number of other areas of clinical investigation). Thus, the field of vitamin D research has the potential to set an example worthy of emulation by researchers in other clinical treatment fields.
